# The Heritability of Replication Problems

**DOI:** 10.3390/cells10061464

**Published:** 2021-06-11

**Authors:** Jean-Sébastien Hoffmann

**Affiliations:** Laboratoire de Pathologie, Laboratoire d’Excellence Toulouse Cancer, CHU Toulouse, Institut Universitaire du Cancer-Toulouse, Oncopole, 1 Avenue Irène-Joliot-Curie, CEDEX, 31059 Toulouse, France; jean-sebastien.hoffmann@inserm.fr

**Keywords:** DNA replication, replicative stress, DNA damage, replication timing

## Abstract

The major challenge of DNA replication is to provide daughter cells with intact and fully duplicated genetic material. However, various endogenous or environmental factors can slow down or stall DNA replication forks; these replication problems are known to fuel genomic instability and associated pathology, including cancer progression. Whereas the mechanisms emphasizing the source and the cellular responses of replicative problems have attracted much consideration over the past decade, the propagation through mitosis of genome modification and its heritability in daughter cells when the stress is not strong enough to provoke a checkpoint response in G2/M was much less documented. Some recent studies addressing whether low replication stress could impact the DNA replication program of the next generation of cells made the remarkable discovery that DNA damage can indeed be transmitted to daughter cells and can be processed in the subsequent S-phase, and that the replication timing program at a subset of chromosomal domains can also be impacted in the next generation of cells. Such a progression of replication problems into mitosis and daughter cells may appear counter-intuitive, but it could offer considerable advantages by alerting the next generation of cells of potentially risky loci and offering the possibility of an adaptive mechanism to anticipate a reiteration of problems, notably for cancer cells in the context of resistance to therapy.

## 1. Introduction

Accurate replication of the human genome in S-phase and faithful segregation of sister chromatids in mitosis are fundamental for the maintenance of chromosome stability from one generation of cells to the next [1]. Cells that are copying their DNA in preparation for division can suffer from ‘replication stress’ (RS) due to various external or endogenous impediments that slow down or stall replication forks, increasing the risk of leaving genomic DNA domains under-replicated [2]. Indeed, when persistent or irreversible fork arrest occurs, the converging fork from a neighboring origin can compensate by replicating all of the DNA up to the stalled fork. This is facilitated by the excessive loading of pre-replicative complexes (Pre-RCs) onto DNA that remain inactive unless RS happens, named ‘dormant origins’ [3]. However, if two converging replication forks are arrested with no dormant origin in between, a double fork stalling (DFS) event occurs with a serious probability of compromised replication and a generation of under-replicated parental DNA [4].

Specific chromosomal domains have a higher propensity for DFSs and include intrinsically hard-to-replicate repetitive sequences which form secondary non-B DNA structures (e.g., centromeres, telomeres, fragile sites), as well as late S-phase replicating regions or loci with a paucity of active and dormant origins [5]. A great proportion of DFS events occur at common fragile sites (CFSs), which are late S-phase replicating regions with a paucity of replication origins, are enriched in large transcription units [6], and are well-documented for their ability to exhibit single-chromatid gaps, breaks, and constrictions on metaphase chromosomes upon RS.

RS is a major cause of pathologies, including cancer, premature ageing, and other disorders associated with genomic instability [7,8]. While severe RS can lead to the activation of checkpoints and trigger cell cycle arrest prior to entering mitosis [9], local low-level RS does not always lead to checkpoint activation and cell-cycle arrest, meaning that cells can proceed through mitosis.

## 2. Progression of Replication Problems into Mitosis

### 2.1. Stimulation of Mitotic DNA Synthesis in Early Mitosis

Cells that have not fully replicated their genomic DNA are found to enter mitosis when an ultimate mechanism to complete DNA replication at these under-replicated loci, called the mitotic DNA synthesis or MiDAS, takes place [10]. In fact, my group reported the first observation of an unexpectedly late DNA synthesis during investigations that aimed to explore the mechanism by which the specialized DNA polymerase eta (Pol η), best known for its ability to perform translesion synthesis through UV-induced damage, contributes to the replication and stability of CFSs [11]. We found that Pol η can be recruited at CFS during S-phase and can facilitate DNA synthesis at non-B structured DNA within these CFS. Pol η-deficient cells were persistently under-replicated at several CFS in mitotic cells and manifested a delayed replication of these regions in early mitosis. This could be achieved by using an original in situ incorporation assay with the thymidine analogue EdU and the detection of EdU spots in Pol η-depleted mitotic cells, as well as in mitotic cells from a patient with the variant form of xeroderma pigmentosum (XPV), a rare, autosomal, and recessive human genetic syndrome associated with *POL H* gene mutations. This late DNA replication MiDAS was then confirmed to take place at broken CFS loci by combining EdU detection with fluorescent in situ hybridization (FISH) in cells exposed to low doses of aphidicolin treatment [12]. The stimulation of this late DNA synthesis process was found to occur in different situations. A two-fold increase in the percentage of cells with MiDAS was recently reported in BRCA2-deficient cells compared to BRCA2-proficient mitotic cells, with an implication of MUS81 nuclease in this process [13]. MiDAS has also been observed at telomeric sequences upon different stresses [14,15].

The switch from normal DNA replication to MiDAS seems to be due to the action of the ubiquitin ligase TRAIP that provokes replisome disassembly in response to uncompleted DNA replication and allows the accessibility of nucleases and repair factors [16]. When cells enter mitosis, stalled forks at CFSs are then cleaved by the SLX4/MUS81/EME1 complex, a limited end resection favors the single-stranded DNA annealing activity of RAD52 into regions of micro-homology, and the subsequent POLD3-dependent conservative DNA repair synthesis takes place. At telomeres, MiDAS also requires RAD52 but not the MUS81-EME1 endonuclease, suggesting that another nuclease might be involved or that MiDAS events do not all require an initial endonuclease cut [15].

Collectively, these observations established that, while the S-phase was conventionally believed to end before the G2-phase of the cell cycle, it can extend almost to the stage of cell division, at least at some difficult-to-replicate chromosomal domains. A successful replication completion by MiDAS could limit the formation of ultrafine anaphase bridges (UFBs) which impede the proper partition and affect the integrity of chromosomes and the transmission of DNA damage to the next generation of cells (see next paragraphs).

### 2.2. Generation of UFB in Mitosis 

When unresolved by MiDAS, late replication intermediates at CFSs following replication stress can lead to physical connections, referred to as ultra-fine DNA bridges (UFBs), between sister chromatids before anaphase that contain a partially denatured, single-stranded or stretched DNA conformation. This affects proper chromosome segregation and results in sister chromatid nondisjunction, a potential source of genome instability [17]. UFBs cannot be stained with DAPI or other commonly used DNA dyes, but can be detected by immunofluorescence staining for proteins such as Bloom’s syndrome helicase (BLM) and replication protein A (RPA), which bind the bridge and are characterized by the presence of twin FANCD2 repair foci [18]. A complex named BTRR, formed by the BLM helicase, topoisomerase IIIa, RMI1, and RMI2, has been documented to resolve UFBs through the unwinding of late replication intermediates, the decatenation of hemicatenanes, or the dissolution of recombination intermediates [19]. Under normal conditions, the majority of UFBs are resolved by late anaphase and telophase. Some persistent UFBs may generate unresolved DNA damage or experience DNA breakage by mechanical stress or cleavage by endonuclease activity. These breaks can, in turn, be converted into 53BP1 containing nuclear structures, called 53BP1 nuclear bodies (NBs), that are thought to shield DNA lesions in G1 daughter cells (see the next paragraph). However, excessive UFB cannot be resolved in a timely manner, and persistently damaged chromosomes, chromatin bridges, and UFBs can suffer detrimental consequences in terms of cell homeostasis and genome stability.

## 3. Consequences of the Inheritability of Under-Replicated DNA in Daughter Cells 

### 3.1. Transmitted DNA Damage Are Protected within 53BP1 NBs in G1 Phase and Resolved in S Phase of Daughter Cells

When not processed during mitosis, under-replicated DNA regions or DNA breaks at CFSs generated by stressed mother cells can be inherited by unstressed daughter cells where they become sequestered and shielded by NBs that contain 53BP1, a large adaptor protein that interacts with the DNA scaffold made of histones and histone-binding proteins involved in DNA double-strand break (DSB) repair [20,21]. 

This is not surprising since to repair DNA breakage during the G1 phase of the cell division cycle, the HR pathway must be actively suppressed to avoid genomic changes. Such HR inhibition can be achieved by 53BP1 in order to block the molecular actors that create single-strand DNA at the break sites, in preparation for recombination [22].

These findings reveal the concept that a response to RS may also occur in otherwise unstressed G1 daughter cells. As for MiDAS, such an inherited response was documented in different stress contexts. For example, a significant increase in the frequency of 53BP1 NBs was observed in cells lacking the MUS81 nuclease or BRCA2, and their frequency was further elevated when the two proteins were concomitantly depleted [13]. A significant increase in the number of spontaneous 53BP1 NBs in G1 was also observed in the absence of p21, as a hallmark of incomplete DNA replication during the previous cell cycle [23].

It has recently been explored how cells deal with inherited under-replicated DNA sequestered in 53BP1-NBs [24]. Live cell tracking of 53BP1-NBs and PCNA revealed that dissolution of the NBs and repair of the lesions require ongoing DNA replication; RAD52 is critical in this process and 53BP1 is required for confining the replication of the lesion sites to late S-phase. Interestingly, dissolution of the 53BP1-NBs at the transmitted DNA damage is coupled with the activation of replication origins by the replication timing factor RIF1. RAD52 was highly enriched at 53BP1-NB sites that were engaged in the dissolution process, and depletion of RAD52 caused 53BP1-NB dissolution defects and repair impairment. Similarly, the depletion of shieldin complex factors, which work downstream of 53BP1 to prevent DNA-end resection, caused unscheduled RAD51 engagement and 53BP1-NB dissolution defects, thereby establishing a fundamental role for RAD52 in the repair of transmitted damages. The depletion of 53BP1 triggered sister chromatid segregation defects and produced pathological recombination intermediates in DNA damage-inheriting cells, showing that the repair of transmitted damages is important for genome stability. In summary, DNA replication can occur after mitosis in 53BP1-NBs and is required for the repair of inherited lesions from the previous cell cycle to guarantee the maintenance of genome stability. It is interesting to note that such a mechanism is performed with the help of RIF1, which is both a RT regulator in S-phase and a repair factor in G1 to favor NHEJ with the help of 53BP1 and the Ssieldin complex. This strongly suggests that repair of transmitted damage could be spatially and temporally coordinated to replicate events in daughter cells by a mechanism which needs to be further explored.

This mechanism, which highlights how 53BP1-NBs restore the loci at risk and limit genome instability after passage through mitosis, also suggests the idea that full genomic DNA duplication may take more than a single cell division cycle.

### 3.2. RS modifies Pre-RC Formation and Spatial Organization of Replication Origins in the Next Generation of Cells

At the exit of mitosis, the transition from highly compact chromatin to a less compact interphase chromatin overlaps with the loading of replication origin licensing factors, in particular the ORC complex, which are essential for executing proper DNA replication. ORC serves as a scaffold for the subsequent association of CDC6 and CDT1, which together coordinate the loading of the MCM2-7 complex in order to form the prereplication complex (pre-RC) required for replication fork formation and activity [25]. Recently, we reported in human cell lines that aphidicolin-induced replication stress in mother cells did not modify the association with chromatin of the ORCs proteins, CDC6 and, CDT1, but can stimulate the loading of MCM2 and p-MCM2 onto the chromatin in G1/S [26].

Replication factories contain clusters of several origins located in physical proximity to each other that fire nearly simultaneously. This organization involves the formation of chromatin loops corresponding to the inter-origin DNA regions, whose size correlates with the length of the replicons, facilitating access of the initiator proteins required to activate origins. We recently reported that RS in mother cells can induce a spatial re-organization of activated origins in G1 daughter cells [26]. Indeed, the length of chromatin loops in nuclei daughter cells emanating from mock-treated and APH-treated mother cells can be monitored by the “fluorescent DNA halo” technique, allowing visualization by means of fluorescence staining of halo formation around insoluble scaffold that results from unwound DNA loops [27]. Cells were synchronized at the end of G1 with L-mimosin, permeabilized with detergent, and depleted of soluble proteins by extraction with high-salt buffers, allowing supercoiled DNA loops to unwind and form halos. A significant reduction in DNA loops was observed in the aphidicolin-released cells [26], suggesting that RS can modify origin selection along the genome in the next cell cycle and corroborate with the observations that the loading of some MCMs onto chromatin of daughter cells was stimulated by RS in the previous cell cycle.

## 4. RS Modifies the Replication Timing at a Fraction of Chromosomal Domains in Unstressed Daughter Cells 

The changes in MCM loading and origin selection in daughter cells may affect the timing of activation of replication origins of a subset of domains in the subsequent S-phase. To test this hypothesis, mother cells were released from aphidicolin and the replication timing (RT) of the next generation of cells was analysed by using human whole genome microarrays [26,28]. Strikingly, in RKO cells, 5% of the genome daughter cells were replicated earlier when the mother cells were exposed to low RS. Notably, a fraction of chromosomal domains that normally replicate in late S were found to replicate in middle S (late-to-middle switches). Importantly, when RT was monitored in the unstressed third cell generation, normal replication timing was restored. These regions with advanced RT do not seem to be linked to 53BP1-NB formation. At the genomic sequence level, they were found to be large regions that share similar features with late replicated regions, such as poor GC content, few constitutive origins, and low gene abundance. Interestingly, these regions were also found to be associated with specific modifications in chromatin structures upon RS. Indeed, when using the assay for transposase accessible chromatin with high-throughput sequencing (ATAC-seq), a method using the hyperactive transposase Tn5 to cut the accessible chromatin with simultaneous ligation of adapters at cut sites for assaying chromatin accessibility genome-wide a significant stimulation by RS in both ATAC-seq peaks strength and coverage within these advanced domains was shown, suggesting that increased chromatin accessibility stimulated by low RS at a fraction of chromosomal domains may explain earlier replication of these domains. Thus far, changes in RT in daughter cells have been observed only in RKO cells. One explanation relies on the nuclear lamina which participates in the chromosome architecture and dynamics within the nucleus, and controls RT of late domains called lamin-associated domains. Indeed, lamin expression was strongly affected in RKO cells compared to other cell lines [26]. A more flexible chromatin organization in RKO cells could render them more permissive to RT changes.

## 5. Conclusions

**Figure 1:**
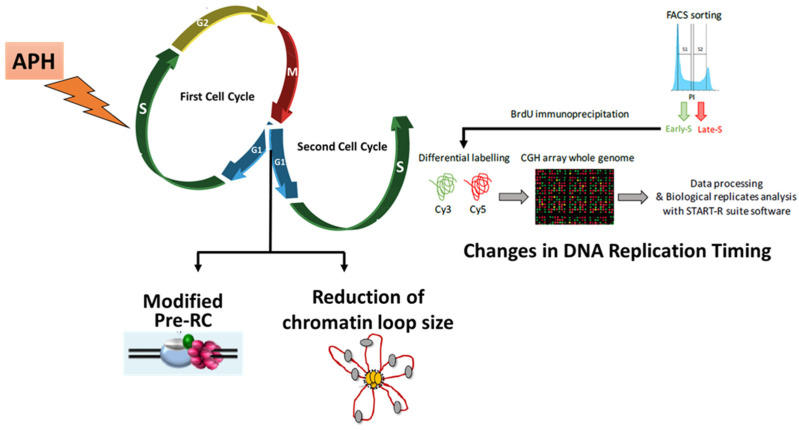
Consequences of RS in the subsequent generation of unstressed cells. Daughter cells showed modified Pre-RC in G1, reduction in chromatin loop size at G1/S transition and changes in DNA replication timing in S phase.

Whereas the mechanisms highlighting the source and the cellular responses of RS have attracted much attention over the last 15 years, the propagation through mitosis of genome modification, and its heritability in daughter cells when the stress is not strong enough to provoke a checkpoint response in G2/M, was much less documented. These recent findings (summarized in Figure 1) led to the conclusion that when mother cells experience low RS, daughter cells are born with reproducible events in G1 after mitosis (53BP1-NBs generation; modified pre-RC formation and spatial organization of origins) and in S-phase (advanced RT within a fraction of large late-replicating chromosomal regions with increased chromatin accessibility). This novel transgenerational response to RS was mostly observed in cancer cell lines. Strikingly, a number of observations indicate that embryonic cells display several signs of replication stress and genomic instability to a level comparable to that observed in cancer cells, most likely due to reduced control of DNA synthesis accuracy and cell cycle checkpoints, which may explain the low rate of successful development in mammals and the occurrence of diseases, such as abnormal developmental features and cancer [29]. It would be interesting to test whether the inheritability of replication problems in embryonic cells is high.

Transmission of replication problems may be an essential step to mark such problematic loci as potentially risky and may thus open up the possibility of a “memory” adaptive response among daughter cells to ensure an efficient replication of these hazardous DNA sequences in the next S-phase. During the clonal evolution of cancer cells, which are often defective in checkpoint and show a high degree of oncogene-induced endogenous replicative stress, such a process may be exacerbated and may contribute to the survival of a cellular clone, especially when exposed to the multiple therapeutic treatments used in clinics that target DNA replication.
